# Exosomal circEhmt1 Released from Hypoxia-Pretreated Pericytes Regulates High Glucose-Induced Microvascular Dysfunction via the NFIA/NLRP3 Pathway

**DOI:** 10.1155/2021/8833098

**Published:** 2021-03-18

**Authors:** Lin Ye, Hui Guo, Yuan Wang, Yun Peng, Yongxin Zhang, Shu Li, Meina Yang, Ling Wang

**Affiliations:** ^1^Shenzhen Eye Hospital, Shenzhen, 518040 Guangdong, China; ^2^Visual-Optic Institute, Health Science Center, Shenzhen University, Shenzhen, 518037 Guangdong, China; ^3^Guangdong Research Institute, Wuhan University, China; ^4^Shenzhen Key Laboratory of Ophthalmology, Shenzhen Eye Hospital, Affiliated Shenzhen Eye Hospital of Jinan University, Shenzhen 518040, China

## Abstract

Diabetic retinopathy (DR) is a frequently occurring microvascular complication induced by long-term hyperglycemia. Pericyte-endothelial cell crosstalk is critical for maintaining vascular homeostasis and remodeling; however, the molecular mechanism underlying that crosstalk remains unknown. In this study, we explored the crosstalk that occurs between endothelial cells and pericytes in response to diabetic retinopathy. Pericytes were stimulated with cobalt chloride (CoCl_2_) to activate the HIF pathway. Hypoxia-stimulated pericytes were cocultured with high glucose- (HG-) induced endotheliocytes. Cell viability was determined using the CCK-8 assay. Western blot studies were performed to detect the expression of proteins associated with apoptosis, hypoxia, and inflammation. ELISA assays were conducted to analyze the release of IL-1*β* and IL-18. We performed a circRNA microarray analysis of exosomal RNAs expressed under normoxic or hypoxic conditions. A FISH assay was performed to identify the location of circEhmt1 in pericytes. Chromatin immunoprecipitation (CHIP) was used to identify the specific DNA-binding site on the NFIA-NLRP3 complex. We found that pericyte survival was negatively correlated with the angiogenesis activity of endotheliocytes. We also found that hypoxia upregulated circEhmt1 expression in pericytes, and circEhmt1 could be transferred from pericytes to endotheliocytes via exosomes. Moreover, circEhmt1 overexpression protected endotheliocytes against HG-induced injury *in vitro*. Mechanistically, circEhmt1 was highly expressed in the nucleus of pericytes and could upregulate the levels of NFIA (a transcription factor) to suppress NLRP3-mediated inflammasome formation. Our study revealed a critical role for circEhmt1-mediated NFIA/NLRP3 signaling in retinal microvascular dysfunction and suggests that signaling pathway as a target for treating DR.

## 1. Introduction

Diabetic retinopathy (DR) is a complication of diabetes that can cause vision loss and blindness in people with diabetes. DR induces serious pathological changes, including thickening of the basement membrane, pericyte apoptosis, endothelial cell dysfunction, increased vascular permeability, and vascular occlusion [1, 2]. As a type of retinal capillary cell, pericytes constitute a class of specialized contractile mesenchymal cells of mesodermal origin that can regulate vascular tone and perfusion pressure by causing the contraction of capillaries [3]. An early loss of pericytes is a hallmark of DR [4] and results in the formation of new capillaries and maturation of a stable vasculature [5–7]. As another type of retinal cell, endotheliocytes are closely linked to pericytes via tight or gap junctions, adhesion plaques, and peg or socket contacts [8, 9]. Normal interactions between pericytes and endotheliocytes are required to establish and maintain homeostasis of the retinal vasculature. Abnormal pericyte-endotheliocyte interactions caused by diabetes can result in retinal vascular leakage and the formation of new leaky vessels [10, 11]. Therefore, it is important to gain a better understanding of the molecular mechanisms that regulate pericyte-endotheliocyte crosstalk. Such insights might help to identify novel targets for preventing and treating DR in a clinical setting.

Circular RNAs (circRNAs) comprise a class of naturally occurring, stable, and ubiquitous RNAs that are formed by the head to tail splicing of coding or noncoding RNAs [12]. Accumulating evidence suggests that circRNAs are important modulators of biological processes and signaling pathways that affect the progression of various diseases, including human cancers [13], neurological diseases [14], and cardiovascular diseases [15–18]. The aberrant expression of several circRNAs (circRNA 0010729 [19], circRNA ZNF609 [20], and circRNA HIPK3 [21]) has been reported in patients with vascular endothelial dysfunction. Liu et al. [22] recently reported that diabetes-related stress upregulates the levels of circRNA cPWWP2A expression in pericytes, which could affect pericyte coverage and vascular integrity by interacting with miR-579 and its target genes, including angiopoietin 1/occludin/SIRT1. Nevertheless, those studies did not elucidate how circRNAs function in mediating pericyte-endotheliocyte crosstalk.

Exosomes are extracellular vesicles that are released from cells upon their fusion with the plasma membrane. Such fusion liberates these intraluminal vesicles into the extracellular milieu, where they can transfer intracellular components such as DNA, RNA, proteins, and lipids from one cell type to another via crosstalk [23]. Under stress conditions resulting from a disease such as DR, the exosome cargo can be altered, and the altered contents might facilitate the development of inflammation [24], glucose starvation [25], and hypoxic/ischemic preconditioning [26]. It is known that pericytes can initiate angiogenesis when cocultured with endothelial cells whose HIF signaling pathway is activated by hypoxia [27]. Because circRNAs are enriched and stable in exosomes and can be transferred to target cells, we speculated that hypoxia-pretreatment of pericytes might alter their exosomal circRNA content, which, in turn, could contribute to the development of high glucose-induced endotheliocyte dysfunction and subsequent aberrant angiogenesis.

To validate our hypothesis, we constructed coculture models of hypoxia pericytes and high glucose-induced endotheliocytes and then used the models to investigate the association between pericyte survival and the angiogenesis activity of endotheliocytes. We then recorded the changes that occurred in the endothelial circular RNA contained in exoxomes released from hypoxia-treated pericytes. Next, we explored the effects of exosomal circRNA derived from hypoxia-pretreated pericytes on high-glucose-induced endotheliocyte injuries. Our results revealed a novel mechanism in which exosomal circRNAs serve as mediators of crosstalk between hypoxia-activated pericytes and high-glucose-induced endotheliocytes to produce retinal microvascular dysfunction.

## 2. Materials and Methods

### 2.1. Cell Culture and Treatments

Murine retinal microvascular pericytes and endotheliocytes were obtained from the American Type Culture Collection (Manassas, VA, USA). To mimic hypoxia, pericytes were cultured until reaching 80–90% confluence and then treated with 200 *μ*M CoCl_2_ (Sigma-Aldrich, St. Louis, MO, USA) for 24 h; at which time, they were designated as hypoxia pericytes. Normoxia pericytes were used as control cells. Next, the hypoxia pericytes were treated with an apoptosis inhibitor (Vad, ApexBio, Houston, TX, USA) or necrostatin-1 (Nec-1, Sigma-Aldrich) for 4 h at 37°C. Endotheliocytes were cultured in Endothelial Cell Medium (ECM, ScienCell, Carlsbad, CA, USA) supplemented with normal glucose (NG; 5.5 mmol/L) as a control or high glucose (HG; 25 mmol/L) under normoxic conditions for 72 h to mimic the early stage of DR. All animal experiments were performed according to Animal Experimental Center of Jinan University.

### 2.2. Coculture Experiments

Pericytes and endotheliocytes were cocultured as previously described [28]. In brief, pericytes were seeded into the wells of a 6-well plate, while endotheliocytes were seeded onto the inner surface of polyethylene terephthalate membranes that were positioned above the pericytes within the same well, without physical cell-to-cell contact. The coculture models were classified into the following groups: (1) normoxia pericytes+endotheliocytes, (2) normoxia pericytes+HG endotheliocytes, (3) hypoxia pericytes+endotheliocytes, (4) hypoxia pericytes+Vad + endotheliocytes, and (5) hypoxia pericytes+Nec-1 + endotheliocytes. The culture medium in each group was replaced with fresh medium every 24 hours. The pericytes and endotheliocytes were separately harvested by trypsinization at the end of the incubation period.

### 2.3. Cell Viability Assay

A Cell Counting Kit-8 kit (CCK-8, Dojingdo Molecular Technologies; Rockville, MD, USA) was used to analyze the viability of pericytes according to the manufacturer's instructions. Briefly, pericytes were seeded into the wells of a 96-well plate at a density of 3,000 cells per well, after 24, 48 h, and 72 h of culture, respectively, 10 *μ*L of CCK-8 solution was added to each well, and the cells were incubated for an additional 2 h at 37°C. The absorbance of each well at 450 nm was measured to assess cell viability.

### 2.4. Pericyte Exosome Isolation and Identification

When the cultured pericytes reached 70–80% confluence, the culture medium was replaced with exosome-depleted FBS, and the pericytes were cultured for another 48 h; after which, they were cultured under either normoxic or hypoxic conditions. After harvesting the culture medium, the normoxia or hypoxia exosomes were isolated using a multistep centrifugation procedure performed as previously described [29, 30]. Following purification in a sterile Ultra-Clear™ tube (Beckman Coulter, Brea, CA, USA), the fractions containing normoxia or hypoxia exosomes were recovered and centrifuged at 4,000 g for 30 min to remove small-cellular debris. The isolated exosomes were then either stored at -80°C or immediately used for subsequent experiments. For identification of exosomes, the exosome pellets were fixed with 2% paraformaldehyde-cacodylate buffer, placed on formvar-covered copper grids, and then further fixed with 1% (*w*/*v*) glutaraldehyde for 5 min. After being washed and dried, the morphology of the isolated exosomes released from pericytes was examined by transmission electron microscopy (TEM). Western blot assays were performed to analyze the expression of specific exosome markers (TSG101, CD63, and CD81).

### 2.5. Exosomal Circular RNA Microarray

A circRNA array pattern analysis of exosomes derived from normoxia or hypoxia pericytes was performed by OE Biotech Company (Shanghai, China). Briefly, exosomal RNAs were isolated using a QIAGEN exoEasy Maxi Kit (QIAGEN GmbH, Germany) and then digested with RNase R (Epicentre Biotechnologie, Inc., Madison, WI, USA) to remove linear RNAs. The obtained enriched circRNAs were labeled using reagents in an Arraystar Super RNA Labeling Kit (Arraystar, Rockville, MD, USA) and then hybridized onto an Arraystar Human circRNAs chip (Arraystar). For microarray analysis, the hybridized arrays were scanned with an Agilent G2565CA Microarray Scanner System (Agilent Technologies, Santa Clara, CA, USA). Agilent Feature Extraction software was used to perform quantile normalization and data preprocessing. Differentially expressed circRNAs were defined as those with a *p* value < 0.05 and *a* > 1.5 fold-change in expression as determined from a comparison between normoxia and hypoxia exosome groups. The results were analyzed by hierarchical clustering.

### 2.6. Circular Characteristics of circEhmt1

The circular characteristic of circEhmt1 was identified by using two experimental methods. Ribonuclease R treatment (RNase R; Duma, Shanghai, China) only degrades linear RNA without affecting circRNA. RNase R treatment entailed extracting RNA from pericytes that expressed exosomes. The pericytes were treated with 3 U/mg RNAse R for 2 h at 37°C; after which, qPCR was used to determine the levels of circEhmt1 and linear Ehmt1 mRNA expression. For divergent primer PCR, cDNA and genomic DNA (gDNA) were extracted from pericytes expressing exosomes using a PureLink™ Genomic DNA Mini Kit (Thermo Fisher Scientific, Waltham, MA, USA). Next, the circEhmt1 and Ehmt1 mRNA were amplified using divergent primers and convergent primers, respectively, with cDNA and gDNA serving as templates.

### 2.7. Exosome Uptake Assay

Exosomes derived from hypoxia pericytes were labeled with the membrane fluorescent red dye PKH26 (CD117, Sigma-Aldrich, USA) according to the manufacturer's protocol. In brief, purified exosomes were resuspended into 1 mL of diluent C and labeled with PKH26. Next, the labeled exosomes were added to endotheliocytes and incubated for 24 h at 37°C. Following incubation, the exosomes were sequentially fixed with 4% paraformaldehyde, rinsed with PBS, counter-stained with DAPI, and finally visualized with a laser confocal microscope (TCS SP5, Leica, Germany).

### 2.8. Fluorescence *In Situ* Hybridization (FISH)

FISH assays were performed to determine the intracellular location of circEhmt1 in pericytes. The FISH assays were performed according to instructions provided by the manufacturer of the FISH kit (RiboBio Co., Ltd., Guangzhou, China). Briefly, pericytes were fixed in 4% formaldehyde, washed with PBS, and then dehydrated through a graded series of ethanol solutions. After incubation with a hybridization buffer, the dehydrated pericytes were mixed with fluorescence-labeled probes for circEhmt1. Next, the nuclei were stained with 6-diamidino-2-phenylindole (DAPI) in the dark, and hybridization signals were visualized with a fluorescence microscope (Leica Microsystems, Germany).

### 2.9. Chromatin Immunoprecipitation (CHIP) Assay

ChIP assays were performed using a Pierce™ Agarose ChIP Kit (Thermo Fisher Scientific) according to the manufacturer's instructions. The primary antibodies used were specific for NFIA (Abcam, Cambridge, MA, USA). Rabbit IgG was used as a mock antibody for a negative control.

### 2.10. Endotheliocyte Treatment and Transfection

EIPA was purchased from Sigma-Aldrich, USA. Lentivirus vectors for circEhmt1 and small interfering targeting NFIA (si-NFIA), as well as a NLRP3 overexpression plasmid and empty negative control vector, were purchased from RiboBio Co., Ltd. (Guangzhou, China). HG-treated endotheliocytes were incubated with hypoxia pericytes expressing exosomes and then divided into the following four groups: (1) HG + Exo + EIPA, (2) HG + Exo + NC, (3) HG + Exo + EIPA+circEhmt1, and (4) HG + Exo + si-NFIA. For the rescue experiments, HG-treated endotheliocytes were transfected with circEhmt1 together with the vector or NLRP3. All transfections were performed for 48 h using Lipofectamine 2000.

### 2.11. Quantitative Real-Time PCR Analysis

Total RNA was extracted using TRIzol (Life Technologies, Carlsbad, CA, USA), and complementary DNA (cDNA) was synthesized using a Bestar™ qPCR RT kit (DBI Bioscience, Ludwigshafen, Germany). Quantitative real-time PCR was performed using Bestar™ qPCR MasterMix (DBI Bioscience) to determine the levels of *HIF*, *Bax*, *Bcl-2*, *circEhmt1*, and *NLRP3* gene expression. The relative expression levels of the above genes were calculated using the 2^−*ΔΔ*Ct^ method, with GAPDH serving as a control. All experiments were performed in triplicate.

### 2.12. Immunofluorescence

Pericytes and endotheliocytes were, respectively, seeded onto coverslips and fixed with 4% paraformaldehyde. Next, the cells were washed with PBS and permeabilized by incubation with 0.5% Triton X-100 at room temperature for 2 h. The cells were then blocked with 5% bovine serum albumin and incubated with Bax primary antibodies (Abcam, Cambridge, MA, USA) overnight at 4°C; after which, the cells were incubated with Alexa Fluor-coupled secondary antibodies for 2 h at room temperature. The nuclei were stained with 4′,6-diamidino-2-phenylindole (DAPI), and images were captured with a fluorescence microscope (LSM510; Zeiss, Toronto, ON, Canada).

### 2.13. Tube Formation Assay

Endotheliocytes were seeded into the wells a 96-well plate (5 × 10^4^ cells per well) that had been precoated with Matrigel Basement Membrane Matrix (BD Biosciences, Franklin Lakes, NJ, USA) and incubated at 37°C for 6 h in normal growth medium. Next, the formation of capillary-like structures was examined and photographed under a light microscope (×200 magnification).

### 2.14. Enzyme-Linked Immunosorbent Assay (ELISA)

The release of inflammatory cytokines, including IL-1*β* and IL-18, into the supernatants of endotheliocytes from different groups was determined using an ELISA kit (R&D Systems, Minneapolis, MN, USA). The concentrations of IL-1*β* and IL-18 are expressed as pictograms per milliliter (pg/mL). All experiments were performed in triplicate.

### 2.15. Western Blot Analysis

The total protein was extracted from whole-cell lysates of pericytes or endotheliocytes using RIPA buffer (Beyotime, Shanghai, China), and the protein concentration in each extract was determined using the BCA assay. A sample of total protein from each extract was separated according to molecular weight on SDS-PAGE gels, and the protein bands were transferred onto PVDF membranes, which were subsequently blocked for 2 h with 5% skim milk. The membranes were then incubated overnight at 4°C with primary antibodies against HIF-1*α*, Bax, Bcl-2, Ki67, TSG101, CD63, CD81, NLRP3, NFIA, ETS-1, and GAPDH. Following incubation, the membranes were washed 3 times with TBST and then incubated with HRP-conjugated secondary antibodies for 2 h at room temperature; after which, the immunostained proteins were detected with enhanced chemiluminescence reagents (GE Healthcare Japan, Tokyo, Japan).

### 2.16. Statistical Analysis

All experiments were performed in triplicate, and results are expressed as a mean value ± standard deviation (SD). All statistical analyses were conducted using GraphPad Prism 6 software. *p* values were calculated using analysis of variance (ANOVA) followed by post hoc Tukey's multiple comparison tests. A *p* value < 0.05 was considered to be statistically significant.

## 3. Results

### 3.1. Pericyte Survival Was Negatively Correlated with Endotheliocyte Angiogenesis

**Figure 1:**
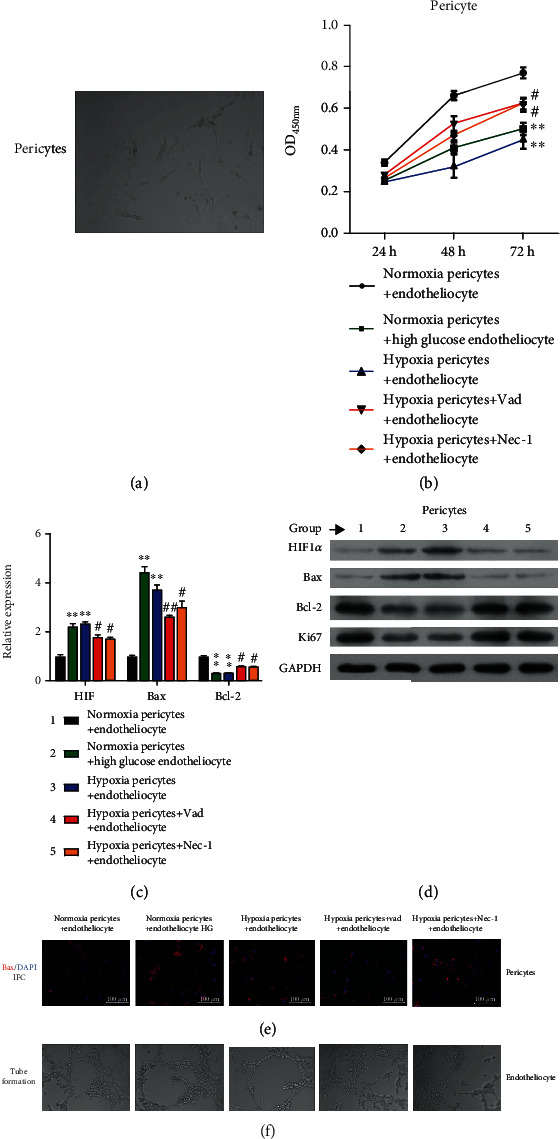
Pericyte survival was negatively associated with endotheliocyte angiogenic activity. (a) The morphology of pericytes cocultured with endotheliocytes. Cocultures of pericytes and endotheliocytes were classified into five groups: (1) normoxia pericytes+endotheliocytes, (2) normoxia pericytes+HG endotheliocytes, (3) hypoxia pericytes+endotheliocytes, (4) hypoxia pericytes+Vad + endotheliocytes, and (5) hypoxia pericytes+Nec-1 + endotheliocytes. (b) The viability of pericytes in the above coculture models was examined. (c) The levels of HIF, Bax, and Bcl-2 mRNA expression in pericytes in the above culture models were determined using quantitative real-time PCR. (d) The levels of HIF-1*α*, Bax, Bcl-2, and Ki67 protein expression in pericytes in the above culture models were detected by western blotting. (e) Immunofluorescence staining of Bax in endotheliocytes in the above coculture models. (f) The angiogenesis activity of endotheliocytes in the above culture models was measured by tube formation assays.

To investigate the interaction that occurs between pericytes and endotheliocytes during the onset of high glucose-induced DR pathogenesis, we cocultured hypoxia pericytes and HG-induced endotheliocytes with or without Vad or Nec-1 treatment. The morphology of the pericytes is shown in Figure 1(a). CCK-8 assays (Figure 1(b)) showed that exposure to either hypoxic conditions or HG treatment decreased the viability of pericytes in the coculture models. Additionally, we found that either Vad or Nec-1 treatment could significantly reverse the decrease in viability of the hypoxia pericytes when the pericytes were cocultured with endotheliocytes under conditions of a normal glucose concentration. Moreover, quantitative real-time PCR (Figure 1(c)), western blot analyses (Figure 1(d)), and immunofluorescence staining for Bax (Figure 1(e)) all demonstrated that the HG-induced hypoxia pericytes had upregulated the levels of both HIF and Bax expression and downregulated levels of Bcl-2 and Ki67 when cocultured with endotheliocytes. Furthermore, the increased levels of HIF, Bax, Bcl-2, and Ki67 in hypoxia pericytes that were cocultured with endotheliocytes were abolished after Vad or Nec-1 treatment. To determine whether pericyte survival affects angiogenesis, we performed tube formation assays using endotheliocytes from different coculture models. As shown in Figure 1(f), endotheliocytes formed greater numbers of tubular structures when the numbers of surviving pericytes were decreased. These data suggested that there was an inverse relationship between pericyte survival and endotheliocyte tube formation.

### 3.2. Exosomal circEhmt1 Was Upregulated in Hypoxia-Pretreated Pericytes

**Figure 2:**
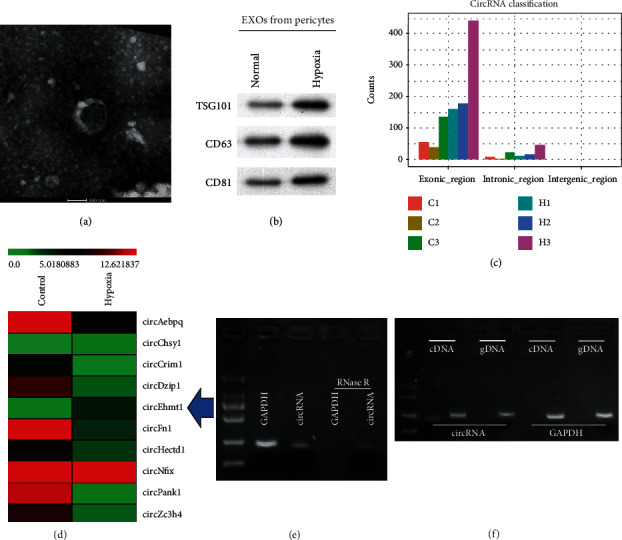
Exosomal circEhmt1 was upregulated in hypoxia-pretreated pericytes. (a) Exosome morphology was observed by TEM. (b) The exosome surface markers TSG101, CD63, and CD81 were analyzed by western blot analysis. (c) All expressed exosomal circRNAs were classified. (d) Heat map representation of the top 10 differently expressed exosomal circRNAs. The circular structure of circEhmt1 was verified by (e) RNase R treatment and (f) divergent primer PCR; cDNA: complementary DNA; gDNA: genomic DNA. C1, C2, C3: samples from control. H1, H2, H3: samples from hypoxia cells.

To explore the mechanism by which pericytes affect angiogenesis, we further investigated the process of endotheliocyte tube formation. Exosomes were isolated from pericytes cultured under hypoxic or normoxia conditions. TEM observations revealed the presence of circular or elliptical vesicles with disc- and cup-shaped structures, which was consistent with the morphological features of exosomes (Figure 2(a)). Western blot analyses further confirmed that the structures were exosomes, based on the presence of exosome signature makers (TSG101, CD63, and CD81), which were more highly expressed in exosomes from hypoxia pericytes than in exosomes from normal pericytes. This difference in exosomes derived from hypoxia and normoxia pericytes further confirmed the successful isolation of exosomes (Figure 2(b)). We next conducted a gene microarray analysis that allowed us to catalogue the exosomal circRNAs that were differentially expressed in hypoxia and normoxia pericytes. As shown in Figure 2(c), the differentially expressed exosomal circRNAs were mainly derived from exons. A hierarchical clustering heat map of the top 10 differentially expressed exosomal circRNAs revealed differences between the exosomes produced by hypoxia and normoxia pericytes (Figure 2(d)). Notably, among all the upregulated exosomal circRNAs, circEhmt1 displayed the largest percentage of upregulation and was therefore selected for subsequent analysis. Prior to conducting further experiments, the circular structure of circEhmt1 was validated by showing it had the highest resistance to degradation by RNase R (Figure 2(e)). Furthermore, only divergent primers were able to amplify its cDNA (Figure 2(f)).

### 3.3. Exosomal circEhmt1 Released from Hypoxia-Stimulated Pericytes Protected Endotheliocytes from HG-Induced Injury

**Figure 3:**
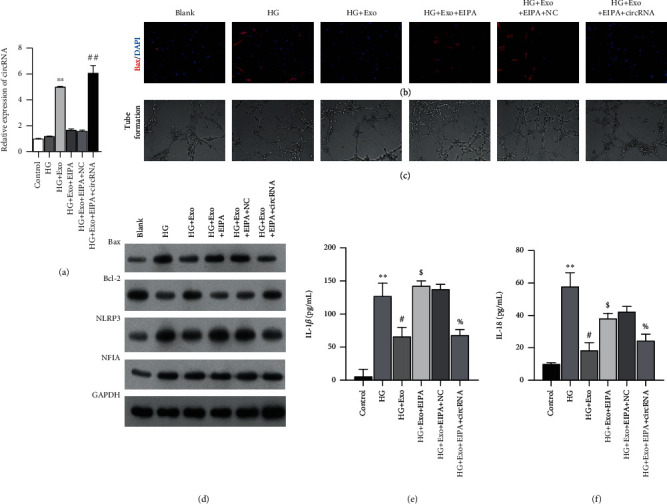
Exosomal circEhmt1 released from hypoxia-stimulated pericytes protected endotheliocytes against HG-induced injury. HG-induced endotheliocytes were incubated with hypoxia-pretreated pericyte exosomes, followed by EIPA treatment with or without transfection with the circEhmt1 overexpression plasmid. (a) circEhmt1 expression in endotheliocytes was determined by quantitative real-time PCR. (b) Immunofluorescence staining of Bax in endotheliocytes. (c) Angiogenesis activity in cultured endotheliocytes was determined by tube formation assays. (d) The levels of Bax, Bcl-2, NLRP3, and NFIA protein expression in endotheliocytes were detected by western blotting. (e, f) The levels of IL-1*β* and IL-18 in endotheliocytes were measured by ELISA. Data are expressed as a mean value ± SD. ^∗∗^*p* < 0.01, compared with control; ^#^*p* < 0.05, ^##^*p* < 0.01, compared with HG; ^$^*p* < 0.05, compared with HG = +Exo; ^%^*p* < 0.05, compared with HG + Exo + EIPA+NC.

Because circEhmt1 was identified as a critical regulator in hypoxia-pretreated pericytes, HG-induced endotheliocytes were incubated with hypoxia-pretreated pericyte exosomes with or without the pinocytosis inhibitor, EIPA. As shown in Figure 3(a), circEhmt1 expression was significantly upregulated in the HG + exosome group when compared with the HG group, whereas EIPA treatment attenuated that increase. Notably, circEhmt1 overexpression obtained via plasmid transfection increased the levels of circEhmt1 expression in the HG + exosome+EIPA group. Immunofluorescence staining for Bax (Figure 3(b)) and results from tube formation assays (Figure 3(c)) indicated an increased release of exosomal circEhmt1 from hypoxia-pretreated pericytes, which led to a decrease HG-induced apoptosis and an increase in HG-induced angiogenesis in cultured endotheliocytes. A western blot analysis showed that exosomal circEhmt1 released from hypoxia-stimulated pericytes downregulated the levels of Bax and NLRP3 expression, but upregulated Bcl-2 expression in HG-induced endotheliocytes (Figure 3(d)). In addition, the release of exosomal circEhmt1 from hypoxia pericytes reduced the levels of proinflammatory cytokines IL-1*β* (Figure 3(e)) and IL-18 (Figure 3(f)) in HG-induced endotheliocytes. These findings suggest that an increase in exosomal circEhmt1 release from hypoxia-pretreated pericytes helps to protect against the effects of HG-induced endotheliocytes by suppressing the generation of NLRP3 inflammasomes.

### 3.4. Exosomal circEhmt1 Reduced HG-Induced Endotheliocyte Injury by Downregulating the NFIA/NLRP3 Pathway

**Figure 4:**
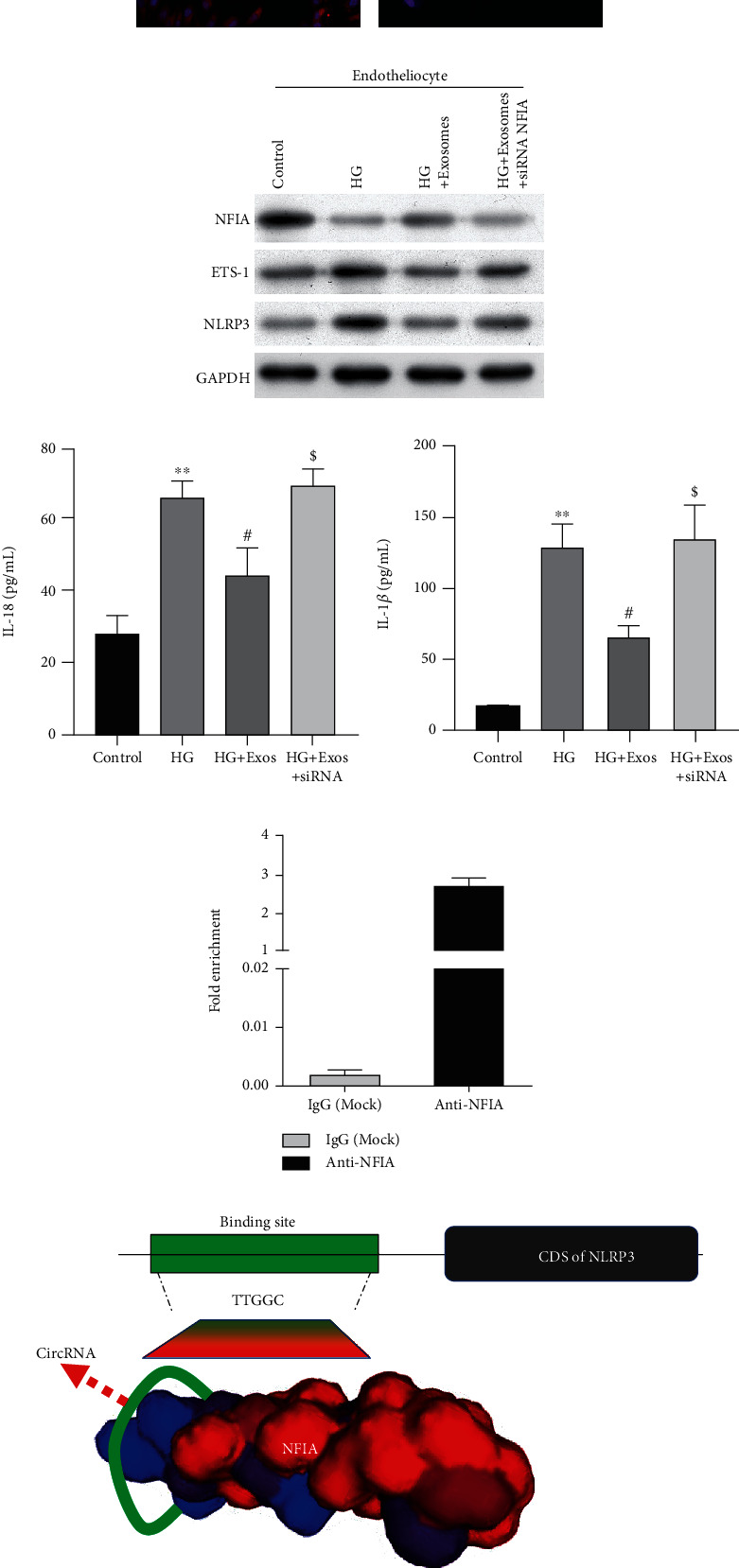
Exosomal circEhmt1 protected endotheliocytes from HG-induced injury by downregulating the NFIA/NLRP3 pathway. (a) Exosomes derived from hypoxia pericytes were labeled with the membrane fluorescent red dye PKH26 and subsequently used in exosome uptake assays. (b) circEhmt1 was mainly expressed in the nucleus of pericytes, as detected by fluorescence after situ hybridization (pink: circEhmt1; blue: DAPI-labeled nuclei; scale bar: 200 *μ*m). HG-induced endotheliocytes were incubated with hypoxia-pretreated pericyte exosomes, followed by si-NFIA transfection. (c) The levels of NFIA, ETS-1, and NLRP3 protein expression in the above-treated endotheliocytes were examined by western blotting. (d) The concentrations of IL-1*β* and IL-18 in the above-treated endotheliocytes were determined by ELISA. Data are expressed as a mean value ± SD. ^∗∗^*p* < 0.01, compared with control; ^#^*p* < 0.05, compared with HG; ^$^*p* < 0.05, compared with HG + exosomes; (e) CHIP assays revealed that NFIA interacted with the promoter region of *NLRP3*. (f) Schematic diagram showing the proposed molecular mechanisms.

To elucidate how exosomal circEhmt1 suppresses HG-induced endotheliocyte injuries, PKH26-labeled exosomes derived from hypoxia-pretreated pericytes were incubated with endotheliocytes. As shown in Figure 4(a), the endotheliocytes effectively incorporated the PKH26-labeled exosomes. Moreover, results of FISH assays showed that circEhmt1 was principally expressed in the nucleus of pericytes (Figure 4(b)). Western blot studies showed that exosomes derived from hypoxia-pretreated pericytes had obviously upregulated levels of NFIA, while ETS-1 and NLRP3 levels were both downregulated in HG-induced endotheliocytes. On the other hand, NFIA knockdown reversed those changes (Figure 4(c)). Results of ELISA assays indicated that NFIA knockdown reversed the declines in IL-1*β* and IL-18 expression in the exosomes produced by HG-treated endotheliocytes (Figure 4(d)). Next, we further confirmed the combined role played by NFIA and NLRP3 in mediating the upregulation of proinflammatory cytokines in HG-induced endotheliocytes. CHIP (Figure 4(e)) experiments consistently demonstrated that NFIA negatively regulated the levels of *NRLP3* gene transcription by binding to the NLRP3 promoter region. Based on our findings, we created a schematic model that shows how circEhmt1 modulates the NFIA/NLRP3 signaling pathway (Figure 4(f)).

### 3.5. NLRP3 Overexpression Reversed the Protective Effect of circEhmt1 against HG-Induced Endotheliocyte Injury

**Figure 5:**
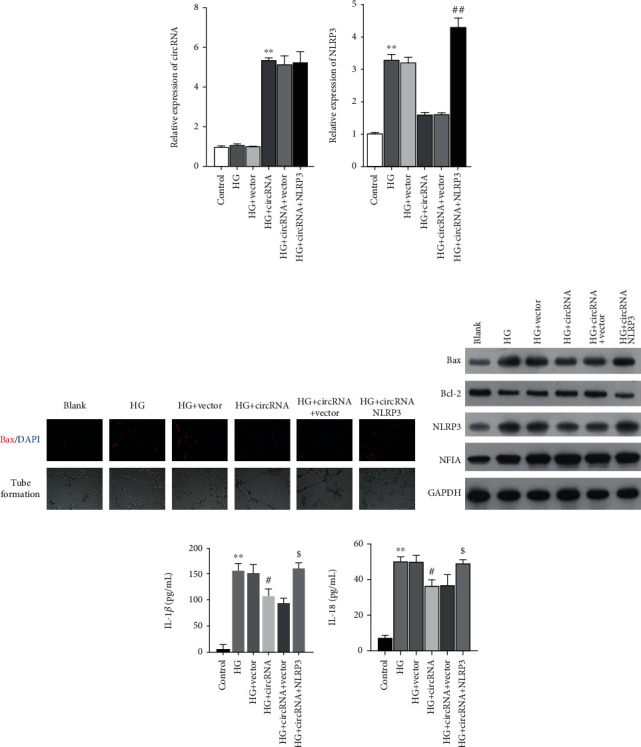
NLRP3 overexpression reversed the protective effect of circEhmt1 against HG-induced endotheliocyte injury. HG-induced endotheliocytes were cotransfected with circEhmt1 and NLRP3 overexpression plasmids. Quantitative real-time PCR was used to determine the levels of circEhmt1 (a) and NLRP3 (b) in HG-induced endotheliocytes. (c) The effects of NLRP3 on the regulatory of circEhmt1 on HG-induced apoptosis and angiogenesis were assessed by immunofluorescence staining of Bax and the tube formation assay, respectively. (d) The levels of Bax, Bcl-2, NFIA, and NLRP3 protein expression in the above-transfected endotheliocytes were detected by western blotting. (e, f) The concentrations of IL-1*β* and IL-18 in the above-transfected endotheliocytes were measured by ELISA. Data are expressed as a mean value ± SD. ^∗∗^*p* < 0.01, compared with control; ^#^*p* < 0.05, compared with HG + vector; ^$^*p* < 0.05, compared with HG + circEhmt1 + vector.

To confirm whether NLRP3 contributes to the circEhmt1-induced attenuation of HG-induced endotheliocyte injuries, rescue experiments were performed using HG-induced endotheliocytes. First, endotheliocytes were cotransfected with circEhmt1 and NLRP3 overexpression plasmids. Quantitative real-time PCR analyses showed that transfection with the cirEhmt1 overexpression plasmid significantly upregulated circEhmt1 expression in HG-induced endotheliocytes (Figure 5(a)), but decreased the levels of NLRP3 expression (Figure 5(b)). Furthermore, overexpression of circEhmt1 reduced HG-induced apoptosis and increased HG-induced angiogenesis activity in cultured endotheliocytes. These effects were attenuated by NLRP3 overexpression, as shown by the results of Bax immunofluorescence staining and tube formation assays (Figure 5(c)). A western blot analysis also showed that NLRP3 overexpression reversed the effects of circEhmt1 overexpression on the levels of Bax, Bcl-2, NLRP3, and NFIA proteins in HG-induced endotheliocytes (Figure 5(d)). In addition, the decreases in IL-1*β* (Figure 5(e)) and IL-18 (Figure 5(f)) levels induced by circEhmt1 overexpression were abolished in HG-induced endotheliocytes.

## 4. Discussion

Here, we first showed that hypoxia simulation with CoCl_2_ decreased the viability of pericytes that were cocultured with HG-induced endotheliocytes. Moreover, pericyte survival was inversely related to endothelial angiogenesis, indicating that an increase in pericyte density could suppress tube formation by endothelial cells. We also found that hypoxia could induce apoptosis in pericytes that were cocultured with HG-induced endotheliocytes, as indicated by an increase in proapoptotic Bax expression and a decrease in antiapoptotic Bcl-2 expression. It is generally believed that the Bcl-2 to Bax protein ratio is indicative of the survival of cells stimulated by apoptotic factors [31]. Hypoxic culture conditions simulated by incubating cells with CoCl_2_ activated the HIF signaling pathway as indicated by an upregulation of HIF-1*α* levels in endotheliocytes. In line with our data, the activation of the HIF signaling pathway has been described as a molecular tool for converting pericytes to a proangiogenic state. Thus, it is not hard to understand that the activation of the HIF pathway leads to a decrease in pericyte survival and a higher level of endotheliocyte angiogenesis, as indicated by an increase in endotheliocyte tube formation.

It is well-known that interactions between endothelial cells and pericytes in the blood vessel wall are essential for regulating retinal vascular formation, function, and remodeling. However, aberrations in pericyte-endotheliocyte crosstalk can lead to vascular dysfunction [4]. The absence of pericytes is a recognized hallmark of DR. In the absence of pericytes, acellular capillaries (i.e., tubes formed by the basement membrane only), capillary occlusions, and microaneurysms (the first clinically relevant lesions in diabetic eyes) occur, together with a later loss of endotheliocytes [32].

Our circRNA expression profiling studies showed that the hypoxia-induced circEhmt1 in pericytes could be transferred from pericytes to endotheliocytes by exosomes. Moreover, we demonstrated that circEhmt1 overexpression could protect endotheliocytes against HG-induced injury *in vitro*, as manifested by decreased levels of cell apoptosis, inflammation, and a suppression of HG-induced increases in angiogenesis activity. The proliferation and recruitment of pericytes are crucial for retinal neovascularization. Disruption of these processes hinders the maturation of new blood vessels and causes them to remain fragile and leaky [33]. Here, we verified that endotheliocytes could effectively uptake PKH26-labeled exosomes derived from hypoxia pericytes. Endotheliocytes usually undergo a phenotypic switch from a normal quiescent phenotype to an apoptotic and active phenotype when responding to hyperglycemic stress [34]. Our data indicated that hypoxia-induced increases in circEhmt1 expression could be caused by exosomes, which are taken up by HG-induced endotheliocytes to suppress their apoptosis and decrease their angiogenic activity. This suggests that circEhmt1-mediated signaling plays an important role in regulating pericyte-endotheliocyte crosstalk. Additionally, the modulation of angiogenesis by exosome delivery of circRNA into endotheliocytes has been reported to be an essential form of crosstalk that enables pericytes to modulate endotheliocyte function [22, 35]. Thus, our study provides a novel insight into how aberrant intercellular communication between endothelial cells and pericytes leads to a loss of blood vessel stability and the development of leaky vessels.

At the molecular level, circRNAs mediate gene transcription by acting as RNA-binding protein sequestering agents or nuclear transcriptional regulators [18]. Our study showed that circEhmt1 is principally expressed in the nucleus of pericytes and upregulates the expression of NFIA, which is a transcription factor that suppresses NLRP3-mediatedinflammasome formation. NFIA knockdown significantly reversed the exosome-induced reductions in IL-1*β* and IL-18 levels in HG-treated endotheliocytes. NFIA, a member of the NFI family, not only plays a major role in regulating lipid droplet formation and astrocyte differentiation [36, 37] but also influences inflammatory responses. An upregulation of NFIA levels was shown to attenuate lipopolysaccharide-induced increases in proinflammatory cytokines such as interleukin- (IL-) 6 and tumor necrosis factor-*α* [38]. Zhou et al. [39] reported that overexpression of NFIA remarkably inhibited endoplasmic reticulum stress and mitochondrial-mediated apoptosis. Consistent with these findings, our study demonstrated that NFIA could suppress *NLRP3* gene transcription by binding to the *NLRP3* promoter. Rescue experiments showed that the inhibition of NLRP3 inflammasome generation could reverse the effect of circEhmt1 on pericyte-endotheliocyte crosstalk and HG-induced retinal vascular dysfunction.

NLRP3 inflammasomes play an important role in diabetes-associated vascular dysfunction. For example, NLRP3 deficiency prevents diabetes-associated vascular inflammatory damage and endothelial dysfunction [40]. Moreover, NLRP3 activation contributes to diabetes-associated vascular dysfunction and a proinflammatory phenotype [41]. Based on these findings, we speculated that the activation of the HIF signaling pathway via the circEhmt1-mediated NFIA/NLRP3 pathway in pericyte exosomes might be a critical mechanism for the pericyte-endotheliocyte crosstalk that affects endothelial angiogenesis.

**Figure 6:**
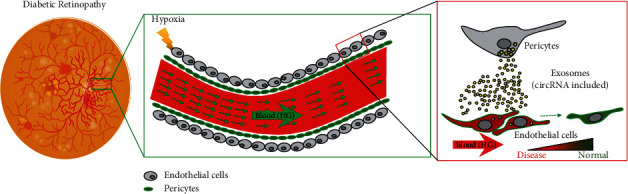
Schematic of this study. Pericytes under hypoxia condition releases exosomes with circular RNA inside. Endothelial cells uptake pericyte-derived exosomes and inflammasomes mediated by NLRP3 were attenuated.

In summary, our present study demonstrated that circEhmt1 was upregulated in pericytes and subsequently transferred from pericytes to endotheliocytes via exosomes. Functionally, circEhmt1 might protect endotheliocytes against HG-induced injury. Mechanistically, circEhmt1 upregulated the levels of a transcription factor (NFIA) that ultimately suppresses the generation of NLRP3 inflammasomes in HG-induced endotheliocytes (Figure 6). Our findings may aid in the development of new clinical strategies for treating DR.

## Data Availability

All data generated or analyzed during this study are included in this article.
